# Simple electroless plating of platinum nanoparticles on graphdiyne for chlorogenic acid electrochemical sensing

**DOI:** 10.1039/d6ra00158k

**Published:** 2026-02-05

**Authors:** Yong Zhou, Geyuan Zheng, Wanlu Chen, Jiang Chen

**Affiliations:** a Implantology Department, School and Hospital of Stomatology, Fujian Medical University Fuzhou 350001 China Dentistjiang@sina.com; b Endodontics Department, School and Hospital of Stomatology, Fujian Medical University Fuzhou 350001 China

## Abstract

Crafting a high-performance electrochemical sensor for chlorogenic acid (CGA) holds substantial value for advancing periodontal disease (PD) management and therapeutic strategies. In this investigation, we synthesized a graphdiyne (GDY)-platinum nanoparticle (Pt NP) hybrid (Pt/GDY) *via* a straightforward electroless plating technique, leveraging the complementary strengths of its components: Pt NPs mitigate GDY aggregation and enhance electrocatalytic activity, while GDY provides large surface and exceptional binding affinity toward CGA. Utilizing this Pt/GDY nanocomposite as the electrode modifier, we engineered an efficient CGA electrochemical sensor. Following optimization of critical detection parameters, including solution pH, Pt/GDY amount and incubation time, the Pt/GDY-based sensor demonstrated outstanding analytical performance, enabling ultrasensitive CGA quantification with a linear range of 0.008–2 µM and a detection limit of 2 nM. Furthermore, the sensor exhibited favorable selectivity against interfering species, long-term stability, consistent reproducibility, and reliable applicability in real samples. These superior characteristics position the Pt/GDY sensor as a promising tool for quality control assessments and drug metabolism studies in PD treatment regimens.

## Abstract 2

In this work, we synthesized a graphdiyne-platinum nanoparticle hybrid *via* an easy electroless plating method and utilized this nanocomposite as the electrode modifier to construct an efficient chlorogenic acid electrochemical sensor.

## Graphical abstract

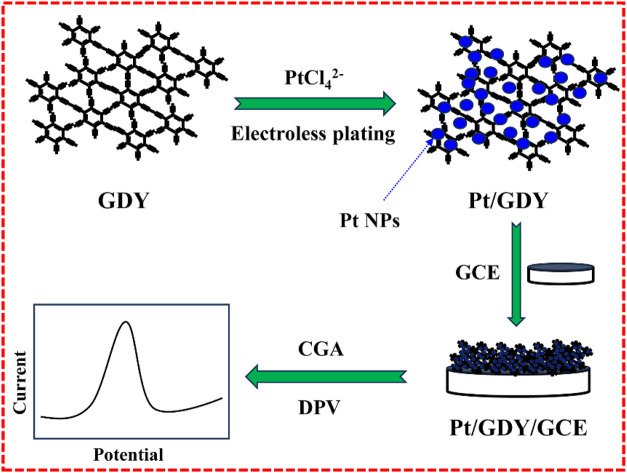

## 1. Introduction

Chlorogenic acid (CGA), a phenolic compound renowned for its low toxicity and minimal adverse effects, is abundantly present in coffee, various fruits, and vegetables.^1–3^ Notably, it serves as the primary bioactive component in numerous traditional Chinese medicinal herbs, such as honeysuckle and Eucommia ulmoides. Extensive research has demonstrated that CGA possesses a broad spectrum of pharmacological benefits, including antioxidant, anti-inflammatory, neuroprotective, and anticancer properties. Of particular relevance to oral health, CGA exhibits significant potential in the prevention and management of periodontal disease (PD).^4–6^ Mechanistically, it promotes the differentiation of human dental pulp stem cells into osteoblasts *via* Wnt signaling pathway modulation, highlighting its promising therapeutic applications for PD patients. Furthermore, CGA's inherent antimicrobial activity positions it as a viable candidate for combating microbe-induced periodontitis, while its incorporation into drug-loaded dental implants has proven effective in reducing infection risks.^7,8^ Consequently, the establishment of rapid, sensitive, and precise analytical methods for CGA detection is crucial for advancing PD intervention strategies and therapeutic monitoring.

Currently, a diverse array of analytical techniques is employed for CGA quantification, encompassing high-performance liquid chromatography, near-infrared spectroscopy, capillary electrophoresis, ultraviolet-visible spectrophotometry, fluorescence assays, and electrochemical methods.^6,9^ Among these, electrochemical approaches stand out as particularly attractive due to their compelling advantages including operational simplicity, exceptional sensitivity, rapid readout and cost-efficiency which facilitate real-time detection of trace-level analytes.^10,11^ However, bare electrodes typically exhibit poor electrocatalytic activity and limited sensitivity toward CGA oxidation,^12,13^ prompting the exploration of advanced nanomaterials as electrode modifiers to overcome these drawbacks and enhance sensor performance.^14,15^ While various innovative nanomaterials have been engineered for CGA electrochemical sensing, achieving effective detection, their synthesis often involves complex preparation protocols. Consequently, developing facile fabrication methods for high-performance CGA electrochemical sensors remains a significant challenge in the field.

**Scheme 1:**
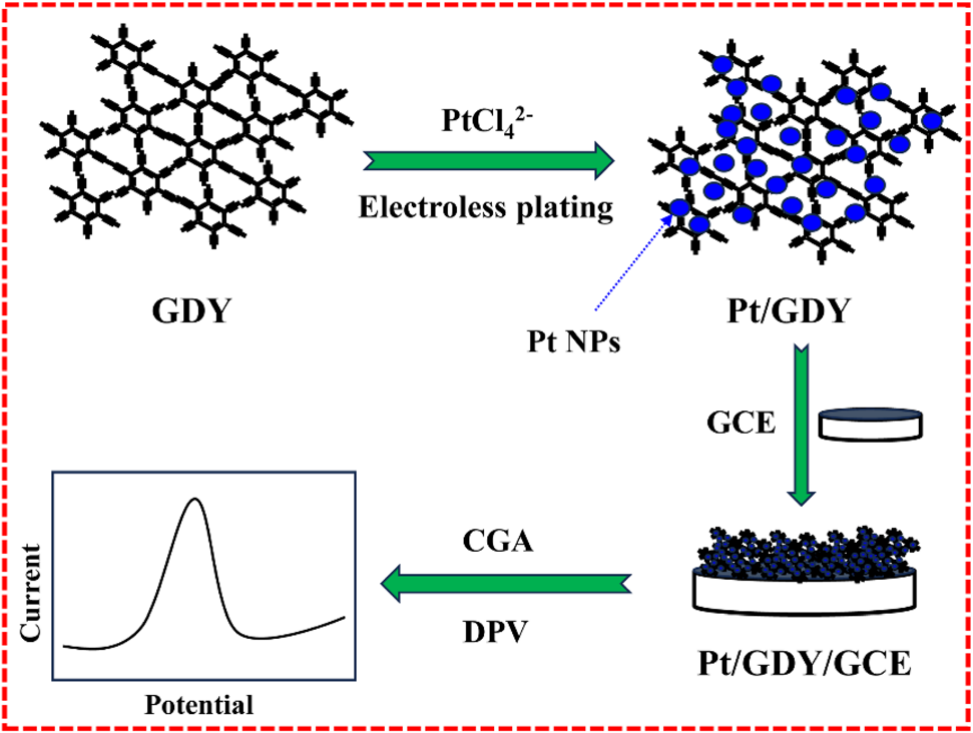
Illustration for the electrochemical detection of CGA by synthesizing Pt/GDY as electrode material based on electroless plating method.

Graphdiyne (GDY), an emerging 2D carbon allotrope composed exclusively of sp/sp^2^-hybridized carbon atoms with benzene rings interconnected by six alkynyl bridges,^16–18^ exhibits a suite of distinctive properties, including exceptional stability, large surface area, conjugated π-electron systems, and excellent conductivity, enabling its versatile application in energy storage, catalysis, and medicinal research.^19–21^ Notably, GDY demonstrates remarkable affinity for target molecules bearing π-electron architectures,^22,23^ prompting its consideration as a prime candidate for developing high-performance CGA electrochemical sensors, given CGA's inherent π-electron structure. However, GDY's pronounced tendency to aggregate poses a significant barrier to its electrochemical sensing applications.^24,25^ Intriguingly, GDY's unique π-electron-rich and alkyne-dense framework endows it with inherent capability to anchor metal nanoparticles (NPs), making some noble metal ions self-reduction to form metal NPs *via* electroless plating, thereby substantially augmenting the composite's catalytic activity.^26–28^ Building on the aforementioned insights, we herein report the synthesis of platinum NPs (Pt NP)-decorated GDY (Pt/GDY) nanohybrids *via* a highly straightforward electroless plating route. The integrated Pt NPs serve multiple critical roles: mitigating GDY aggregation, enhancing its electronic conductivity and catalytic efficacy. By capitalizing on GDY's superior properties especially the intrinsic affinity for π-electron-rich targets like CGA, we engineered a sensitive electrochemical sensor using Pt/GDY as the electrode modifier (Scheme 1). Performance evaluations demonstrated that the Pt/GDY sensor achieves ultrasensitive CGA detection with a low detection limit (LOD) of 2 nM and a broad linear range spanning 0.008–2 µM, alongside excellent anti-interference capability and long-term operational stability. Furthermore, rigorous real-sample analysis validated the sensor's high accuracy, underscoring its substantial potential for practical applications in CGA quantification.

## 2. Experimental section

### 2.1 Chemicals and apparatus

CGA, ascorbic acid (AA), chicoric acid (ChA), uric acid (UA), caffeic acid (CA), catechol (CC), glucose (Glu), and K_2_PtCl_4_ were procured from Sigma-Aldrich Co. (St. Louis, MO, USA). GDY was produced following a reported previously protocol with modifications to optimize yield and structural uniformity.^29^ All reagents were of analytical grade and utilized without additional purification steps to ensure experimental consistency. Ultrapure water (18.2 MΩ·cm) served as the solvent throughout the study unless specified otherwise. Electrochemical measurements were performed using a CHI 760E electrochemical workstation (Chenhua Instruments, Shanghai, China) configured with a common 3-electrode setup: a nanomaterial-modified glassy carbon electrode (GCE) as working electrode, a calomel electrode (Ag/AgCl) as the reference electrode, and a platinum wire as the counter electrode, with saturated KCl solution employed as the reference electrolyte.

### 2.2 Synthesization of Pt/GDY

The Pt/GDY nanohybrid was fabricated *via* a simplified electroless plating protocol:^27^ 5 mL of 0.2 M PtCl_4_^2−^ aqueous solution was gradually introduced dropwise into 30 mL of GDY dispersion (1 mg mL^−1^), followed by continuous magnetic stirring at room temperature for 10 minutes. The resulting Pt/GDY composite can be obtained just through conventional washing and drying steps.

### 2.3 Fabrication of sensor

The sensor of Pt/GDY modified GCE (Pt/GDY/GCE) was fabricated *via* dropping uniform Pt/GDY suspension (12 µL, 0.5 mg mL^−1^) on GCE surface. Meanwhile, GDY/GCE sensor was also fabricated with the same steps for comparison *via* using pure GDY instead of Pt/GDY.

## 3. Results and discussion

### 3.1 Characterization of Pt/GDY

**Fig. 1:**
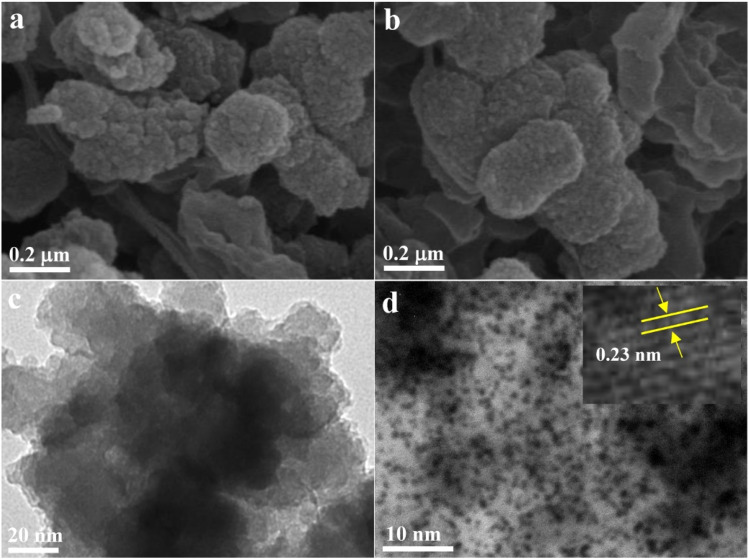
(a and b) SEM and (c and d) TEM images of (a and c) GDY and (b and d) Pt/GDY. The inset of Fig. 1d is the related HRTEM image.

The morphology and crystal structure of the as-prepared Pt/GDY nanohybrid were systematically characterized *via* electron microscopy and spectroscopic techniques. Scanning electron microscopy (SEM, Fig. 1a and b) analysis revealed that Pt/GDY retains the block-like multilayer architecture of pristine GDY, with no significant alteration in overall morphology, confirming that Pt NP deposition does not disrupt the intrinsic structural integrity of the GDY matrix. Fig. S1 displayed the higher-resolution SEM image of Pt/GDY, there is no obvious nanoparticles observed, indicating the Pt NP size is extremely small. Transmission electron microscopy (TEM, Fig. 1c and d) images demonstrated uniformly dispersed Pt NPs (average diameter ∼2 nm, Fig. S2) across the GDY surface, in contrast to the aggregated tendency of bare GDY nanosheets. To further verify the nanoparticles, HRTEM image was performed subsequently. As shown in the inset of Fig. 1d, Pt/GDY exhibits a crystal structure, and the lattice distance of 0.22 nm is attributed to the Pt (111) plane. Inductively coupled plasma-atomic emission spectrometry quantified the Pt loading in the hybrid material as 8.3 wt%. Fourier-transform infrared (FT-IR) spectroscopy (Fig. S3) identified characteristic alkynyl stretching vibrations at 2151 and 2305 cm^−1^ for pure GDY; these peaks underwent distinct deformation in Pt/GDY, attributed to alkyne group consumption and interfacial interactions between GDY and Pt during the self-reduction of Pt(II) ions.^26–28^ Raman spectroscopy of Pt/GDY (Fig. S4) exhibited two prominent peaks: the *D* band and *G* band. Notably, the D/*G* intensity ratio of Pt/GDY (0.82) was significantly higher than that of pure GDY (0.68), indicating that Pt NP incorporation induces a substantial increase in surface defects and active sites—features anticipated to enhance electrochemical sensing performance.

Subsequently, electrochemical impedance spectroscopy (EIS) was employed to evaluate the electron transfer properties of electrodes modified with different nanomaterials, with measurements conducted in a 10 mM K_3_[Fe(CN)_6_] solution containing 0.1 M KCl as the supporting electrolyte (Fig. S5). Nyquist plots revealed that the semicircle diameter of GDY/GCE was slightly larger than that of the bare GCE, indicating moderate electron transfer resistance introduced by GDY coating. In striking contrast, the Pt/GDY-modified electrode exhibited a significantly reduced semicircle diameter compared to GDY/GCE, confirming that the *in situ* grown Pt nanoparticles effectively enhance the conductivity of the GDY matrix by facilitating interfacial electron transport.

### 3.2 Electrochemical sensing of CGA

**Fig. 2:**
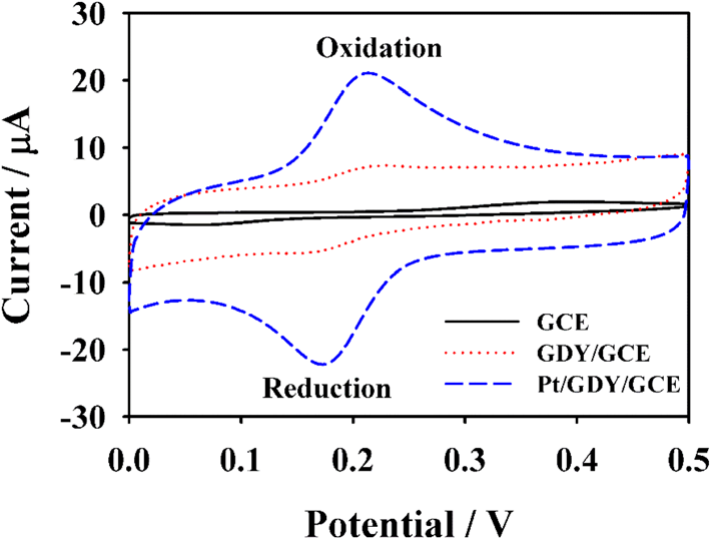
CV responses of 5 µM CGA at bare GCE, GDY/GCE and Pt/GDY/GCE, respectively.

**Fig. 3:**
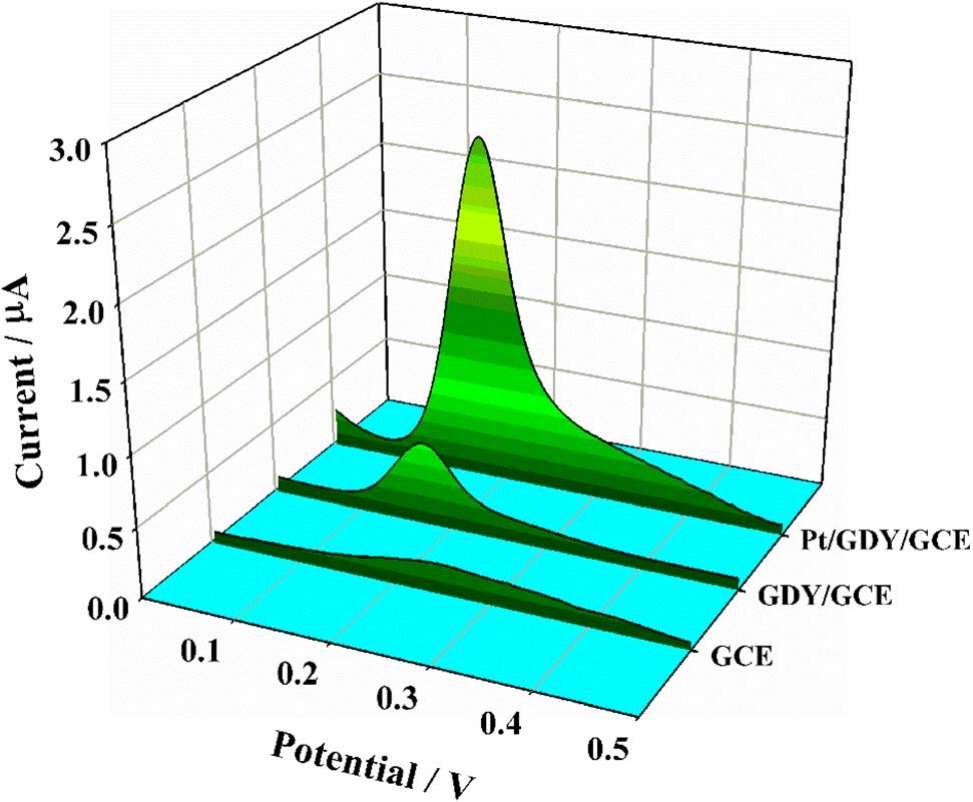
DPV responses of CGA with low concentration (0.2 µM) at bare GCE, GDY/GCE and Pt/GDY/GCE, respectively.

To evaluate the electrochemical sensing performance of the synthesized Pt/GDY toward CGA, we compared the redox behaviors of CGA at electrodes modified with different nanomaterials. Cyclic voltammetry (CV) measurements (Fig. 2) revealed minimal CGA redox peaks at the bare GCE (Oxidation, ∼0.38 V; Reduction, ∼0.17 V), whereas distinct peak signals were observed at GDY/GCE—confirming GDY's ability to amplify CGA electrochemical responses. Notably, the Pt/GDY/GCE sensor exhibited significantly enhanced peak currents (∼3.6-times higher) coupled with a great peak potential shift compared to GDY/GCE, attributed to the synergistic catalytic effect of Pt NPs. Differential pulse voltammetry (DPV), a more sensitive technique than CV, further validated these findings (Fig. 3): Pt/GDY/GCE not only reproduced the CV trends but also detected low-concentration CGA with clear peak currents, highlighting its superior sensitivity. These results collectively demonstrate that Pt/GDY possesses excellent catalytic activity for CGA redox due to Pt NP decoration, making it a promising electrode modifier for CGA sensing applications. The electrochemical redox mechanism of CGA (Fig. S6) involves a reversible two-electron/two-proton transfer process: the catechol moiety (ortho-OH groups on the aromatic ring) oxidizes to form an ortho-quinone, which is subsequently reduced back to the catechol form. The outstanding performance of Pt/GDY arises from dual synergistic effect: (i) GDY's highly conjugated π-electron framework facilitates strong CGA adsorption *via* π–π interactions, and (ii) Pt NPs not only mitigate GDY agglomeration and enhance conductivity but also provide abundant active sites for the electro–catalytic reaction of CGA.

**Fig. 4:**
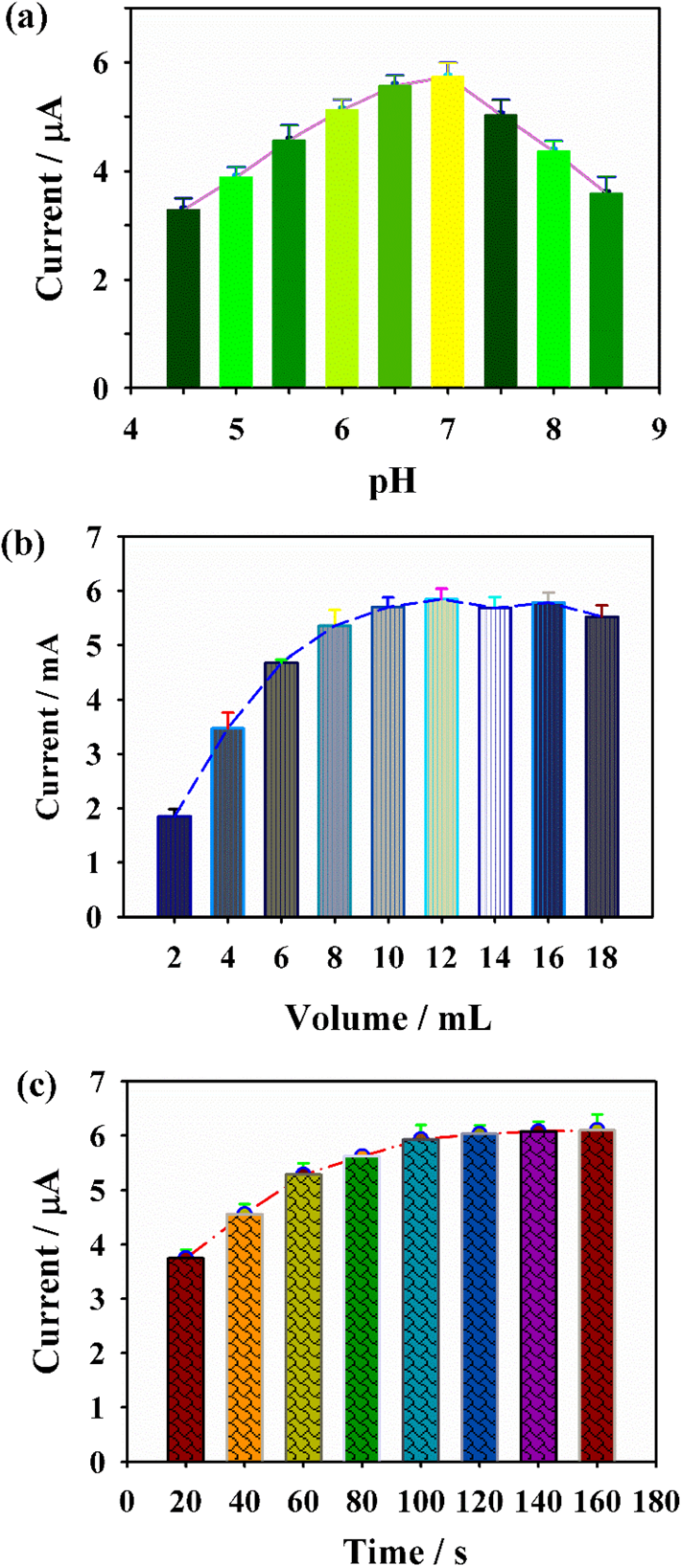
The influences for the Pt/GDY/GCE sensing performances from the (a) pH value of PBS, (b) Pt/GDY amount, and (c) accumulation time.

Subsequently, DPV was employed to systematically optimize key parameters influencing the sensing performance of Pt/GDY/GCE. As shown in Fig. 4a, the current response of Pt/GDY/GCE toward CGA increased progressively with solution pH in the range of 4.5–7.0, followed by a decline at pH > 7.0—likely due to the deprotonation of CGA's catechol groups under alkaline conditions—thus pH 7.0 was selected as the optimal electrolyte pH. The effect of Pt/GDY modification amount was evaluated by varying the droplet volume from 2 µL to 18 µL (Fig. 4b): the DPV peak current increased linearly with volume up to 12 µL, after which it plateaued, indicating saturated surface coverage of the electrode; accordingly, 12 µL was chosen as the optimal modification dosage. Additionally, the impact of accumulation time on CGA adsorption was investigated (Fig. 4c): the current increased gradually from 20 s to 120 s, but showed negligible change between 120 s and 160 s, confirming that CGA adsorption on Pt/GDY/GCE reached equilibrium within 120 s. Collectively, these optimization results established the optimal sensing conditions as pH 7.0, 12 µL Pt/GDY modification, and 120 s accumulation time.

**Fig. 5:**
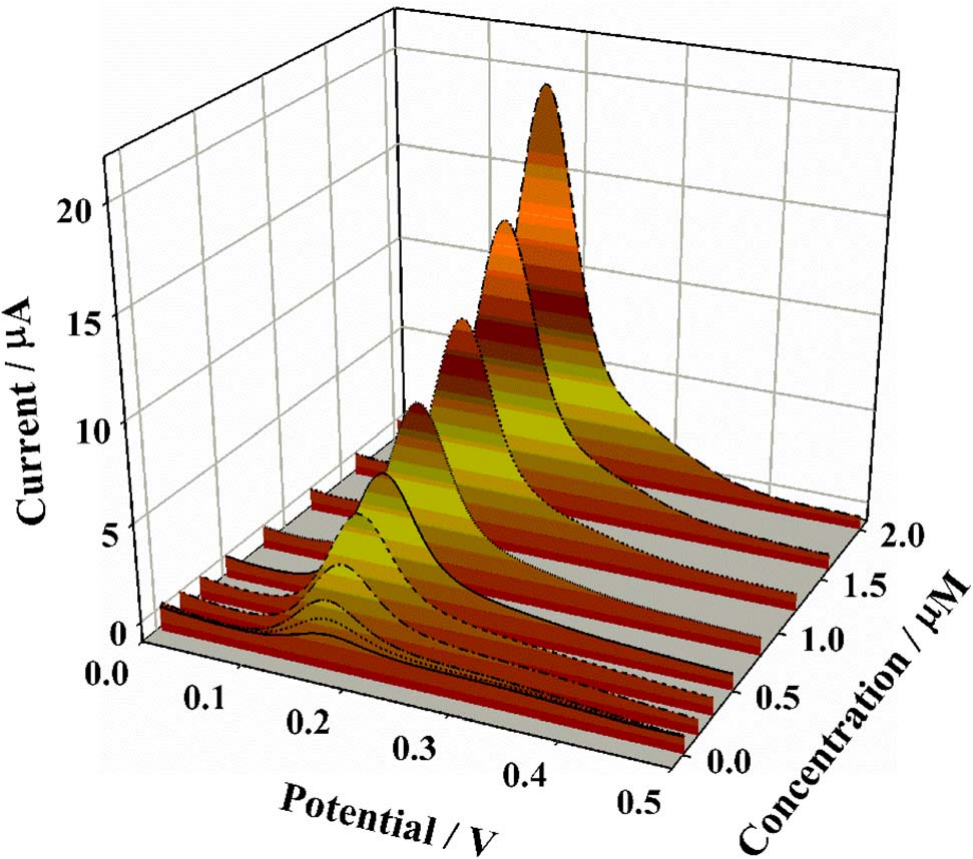
DPV curves of Pt/GDY/GCE in PBS solution containing various concentrations (0.002–2 µM) of CGA.

**Fig. 6:**
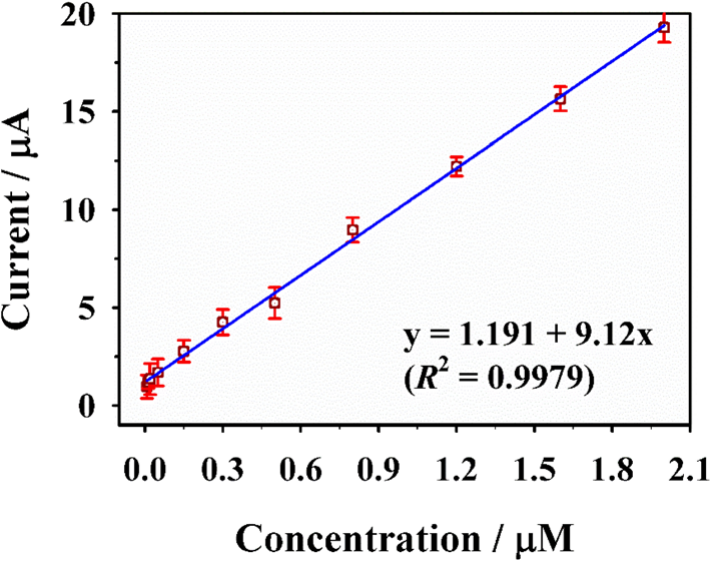
The corresponding calibration curve of the response current *versus* the concentration of CGA.

Under the optimized experimental conditions, DPV was employed to quantify CGA using the Pt/GDY/GCE sensor, revealing a linear relationship between the peak current and CGA concentration in the range of 0.008–2 µM (Fig. 5). The linear regression equation was determined as *y* = 1.191 + 9.12*x* (*R*^2^ = 0.9979), indicating excellent linearity (Fig. 6); the LOD, calculated based on the 3*σ* method, was as low as 2 nM. To further validate the analytical superiority of the Pt/GDY sensor, a comparative analysis with previously reported CGA detection platforms was conducted (Table 1). Notably, the Pt/GDY/GCE exhibited a lower LOD and a comparable or wider linear range than most existing sensors, including those based on metal oxides, carbon nanomaterials, and other hybrid composites, thus demonstrating its exceptional sensitivity and broad detection capability for CGA quantification.

**Table 1:** Comparisons of analytical parameters for detection of CGA among previously described electrochemical sensors and the obtained Pt/GDY/GCE sensor in this work

Materials	Linearities	LODs	References
Fe_2_O_3_-E	0.05–668 µM	6.6 nM	30
Fe-NC	0.05–70 µM	5.2 nM	31
MIL-101(Cr)/MWCNTs	0–14.4 µM	19.8 nM	3
Au/PcFe@HZIF-8	0.03–500 µM	10 nM	32
AuPd/MWCNTs/Ni-ZIF-8	0.1–1 µM	33 nM	33
Pt@Pd NWs-Hemin-PEI-rGO	0.5 mM – 4 mM	7.8 nM	34
ZIF-67/PEDOT/CP	0.1–40 µM	3 nM	10
DSiFPC	0.03–1 µM	6.2 nM	35
PdZn/NP@C	0.005–7.0 µM	1.2 nM	36
Pt/GDY	0.008–2 µM	2 nM	This work

### 3.3 Stability, repeatability, and interference immunity studies

**Fig. 7:**
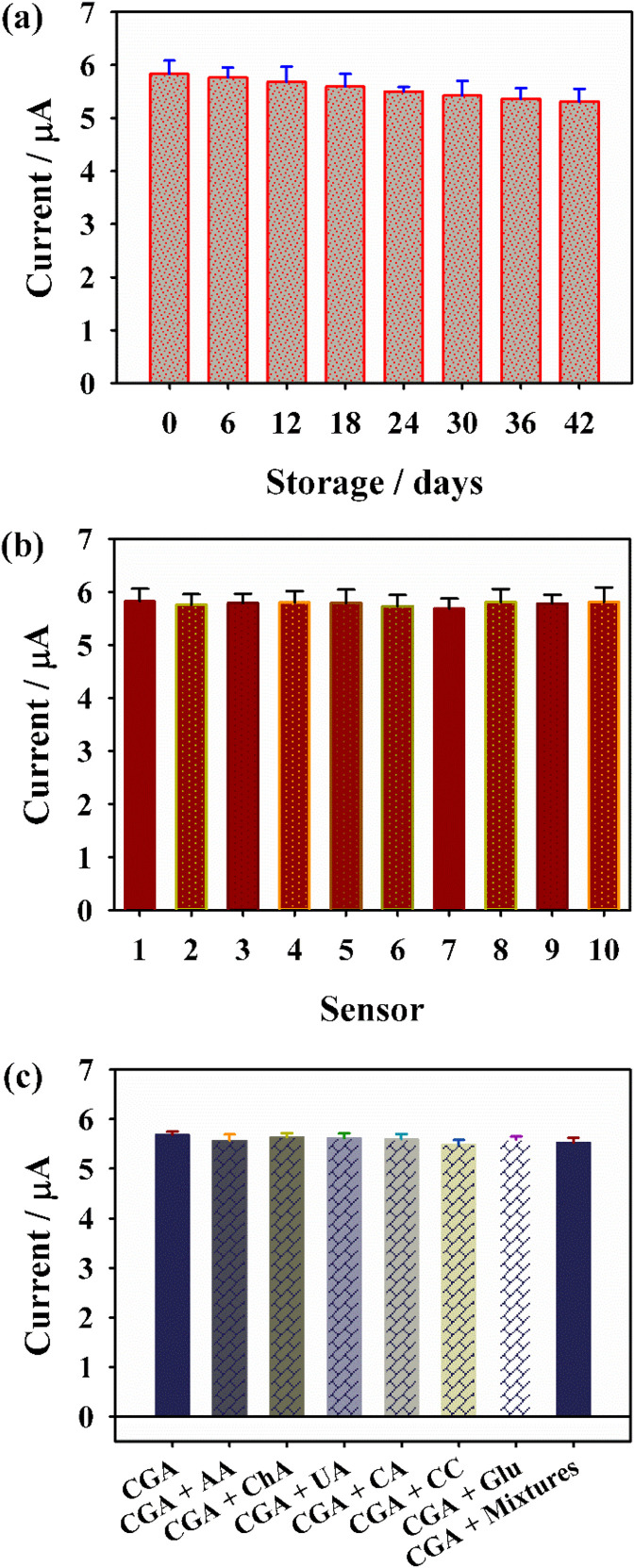
The (a) stability, (b) repeatability and (c) selectivity of sensor.

In this section, critical analytical parameters for real-world applications including long-term stability and reproducibility were systematically evaluated. For stability assessment, the sensor was stored at 4 °C and periodically subjected to DPV measurements in 0.1 M PBS containing 500 nM CGA at 6-day intervals; notably, after 42 days of storage, the peak current response retained 91.2% ± 0.3% of its initial value, confirming robust storage stability (Fig. 7a and S7). Reproducibility was assessed *via* ten independent electrode fabrication, yielding consistent response signals with a relative standard deviation (RSD) of 3.56% (Fig. 7b)—a value well below the typical threshold for analytical sensors, thus demonstrating exceptional fabrication uniformity and measurement precision of the Pt/GDY-based platform.

Evaluating the anti-interference capability of the Pt/GDY sensor is critical for ensuring reliable electrochemical detection of CGA in complex matrices. To characterize its selectivity, DPV was employed to monitor the sensor's response to 500 nM CGA in pH 7.0 buffer, with potential co-interferents including biological molecules (AA, ChA, UA, CA, CC, and Glu) introduced at 10-fold molar excess relative to CGA. As shown in Fig. 7c, the addition of these interferents did not induce significant changes in the peak current or shape compared to the CGA-only system, indicating minimal non-specific interactions with the Pt/GDY interface. Collectively, these results validate the sensor's robust anti-interference performance, supporting its applicability for accurate CGA quantification in real samples with complex compositions.

### 3.4 Application in real samples

To validate the practical applicability of the Pt/GDY sensor for real-world scenarios, CGA quantification was performed in two complex matrices—commercial coffee extracts and human blood serum—using the standard addition method under the optimized experimental conditions. Each sample was analyzed in triplicate to ensure measurement reproducibility, with the quantitative results summarized in Table S1. The sensor exhibited distinct electrochemical responses to spiked CGA concentrations in both matrices, yielding recovery rates ranging from 91.5% to 97.2%. These findings confirm that the Pt/GDY-based electrochemical platform can accurately quantify CGA in real samples despite potential background components, highlighting its promising utility for real samples.

## 4. Conclusions

In conclusion, this study presents a facile yet highly sensitive electrochemical platform for CGA detection, enabled by a Pt/GDY nanohybrid-modified GCE. Synthesized *via* a straightforward electroless plating method, the Pt/GDY nanocomposite synergistically integrates GDY's superior properties and affinity for CGA with Pt NPs multiple roles in mitigating GDY agglomeration, boosting both conductivity and catalytic activity of the modified electrode. Under optimized experimental conditions, the Pt/GDY/GCE exhibits exceptional analytical performance, featuring a low LOD of 2 nM and a wide linear range of 0.008–2 µM for CGA quantification. Complementing these metrics, the sensor demonstrates robust selectivity against common interferents, good reproducibility (RSD = 3.56%, *n* = 10), long-term storage stability (91.2% current retention after 42 days), and reliable real-sample applicability in coffee extracts and serum (recovery rates: 91.5–97.2%). Collectively, these findings validate the Pt/GDY nanohybrid as a promising electrode material for constructing practical electrochemical sensing platforms, with significant potential for applications in clinical diagnostics and food quality monitoring.

## Author contributions

Yong Zhou: writing – original draft, methodology, data curation. Geyuan Zheng: review & editing, methodology, conceptualization. Wanlu Chen: review & editing, conceptualization. Jiang Chen: methodology, supervision, review & editing,.

## Conflicts of interest

There are no conflicts to declare.

## Supplementary Material

RA-016-D6RA00158K-s001

## Data Availability

Data used in this study will be made available upon request from the corresponding author. Supplementary information: the higher-resolution SEM image, the Pt NPs size distribution**,** FT-IR spectroscopy, Raman spectroscopy, EIS plots of different electrodes, electrochemical redox mechanism of CGA, sensor stability and real application results. See DOI: https://doi.org/10.1039/d6ra00158k.
