# Supplementary material for: Simple electroless plating of platinum nanoparticles on graphdiyne for chlorogenic acid electrochemical sensing
Source: RSC Adv. 2026 Feb 5;16(8):7574–80. doi: 10.1039/d6ra00158k (PMC12875311; doi:10.1039/d6ra00158k)
Supplement: RA-016-D6RA00158K-s001 [file RA-016-D6RA00158K-s001.pdf]

Supplementary Information (SI) for RSC Advances.
This journal is © The Royal Society of Chemistry 2026

Supporting information

Simple electroless plating of platinum nanoparticles on graphdiyne for chlorogenic acid electrochemical sensing

Yong Zhou,[a#] Geyuan Zheng,[a#] Wanlu Chen,[b] Jiang Chen[a*]

[a] Implantology Department, School and Hospital of Stomatology, Fujian Medical University, Fuzhou 350001, China

[b] Endodontics Department, School and Hospital of Stomatology, Fujian Medical University, Fuzhou 350001, China

[#] Co-first authors.
[*] Corresponding author. E-mail address: Dentistjiang@sina.com.

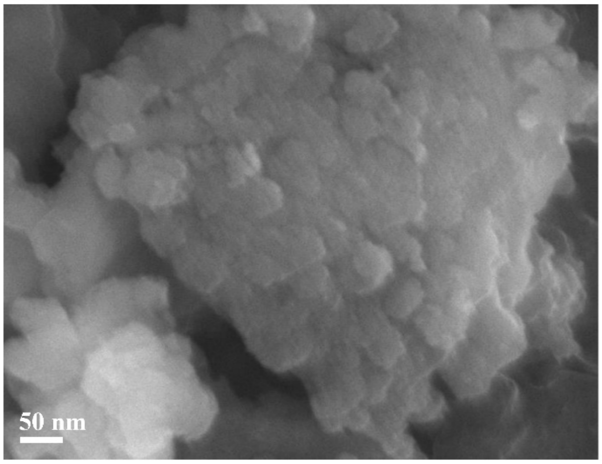

Figure S1. The higher-resolution SEM image of Pt/GDY.

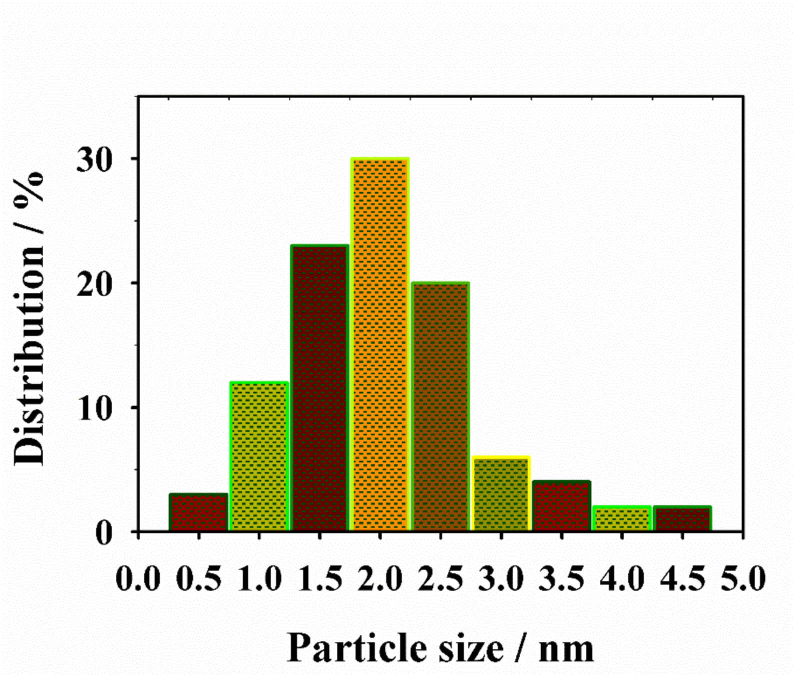

Figure S2. The distribution of Pt NPs size.

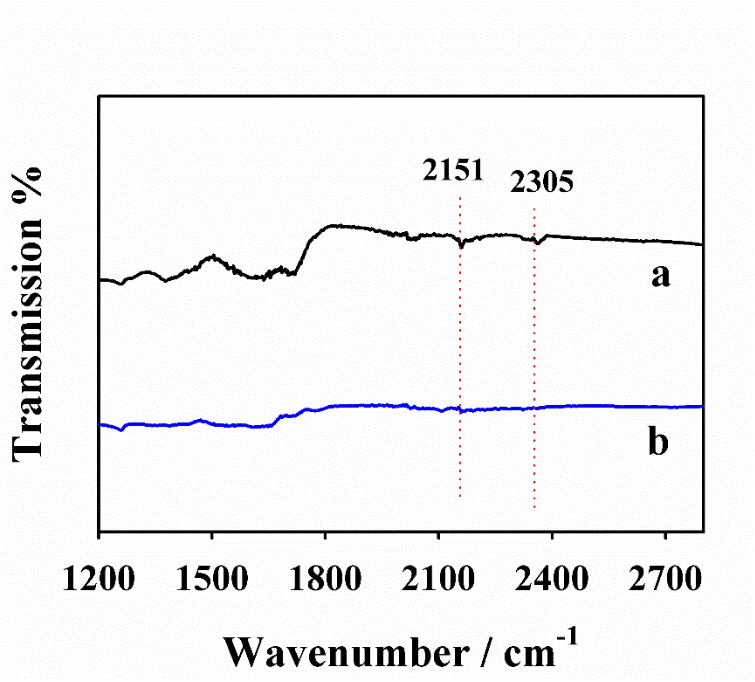

Figure S3. FT-IR spectroscopy of (a) GDY and (b) Pt/GDY.

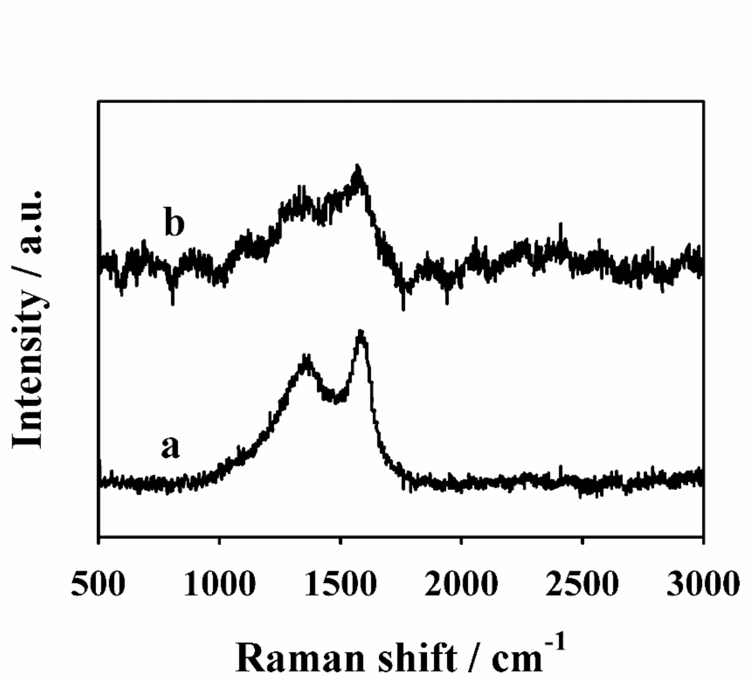

Figure S4. Raman spectroscopy of (a) GDY and (b) Pt/GDY.

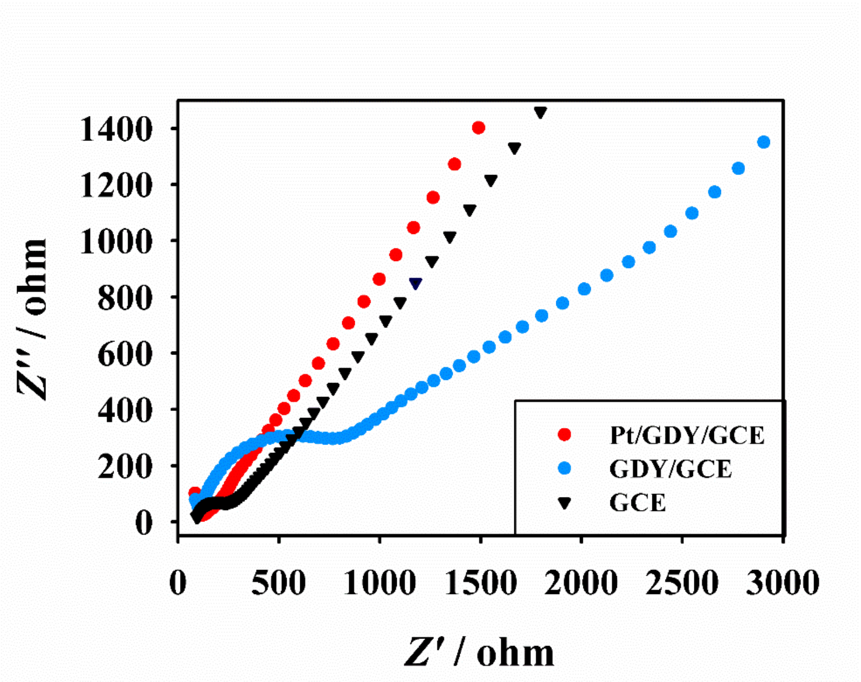

Figure S5. EIS plots of different electrodes.

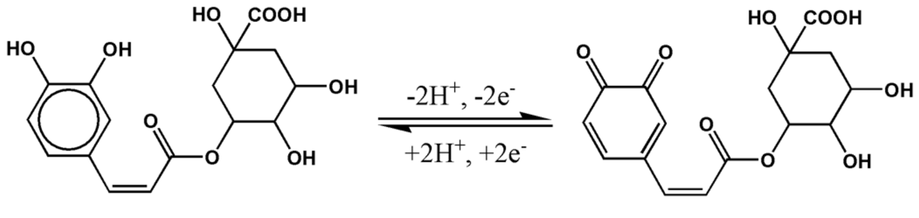

Figure S6. The proposed electrochemical redox mechanism of CGA at Pt/GDY/GCE.

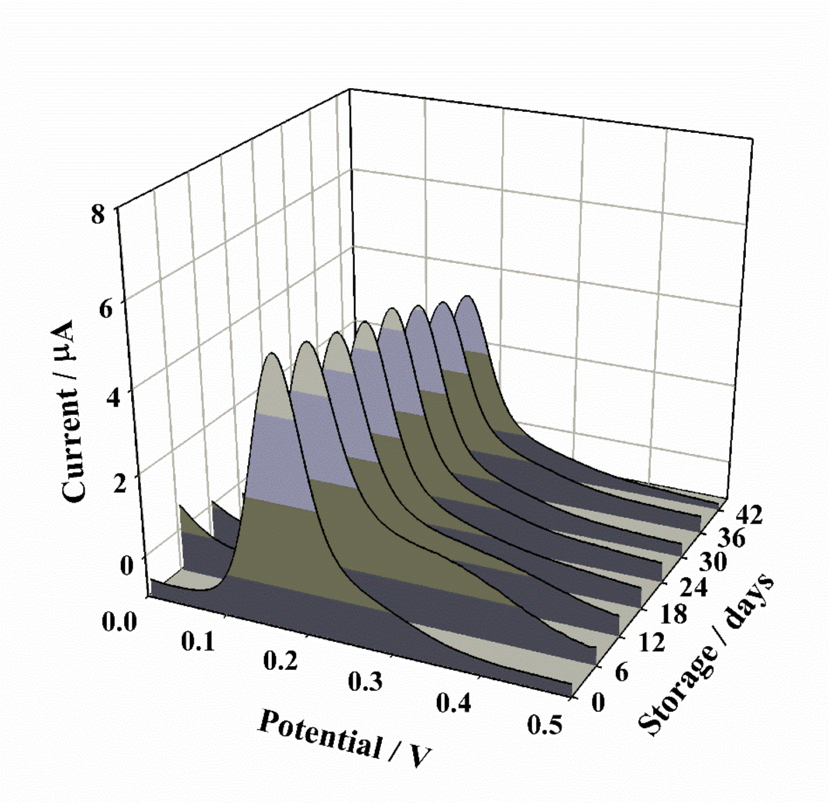

Figure S7. The DPV responses of Pt/GDY/GCE upon different storage time.

Table S1. The detection of CGA based on Pt/GDY sensor in real samples.

Samples	Added / nM	Found / nM	Recovery (%)
Serum	50	48.6	97.2
	200	185.3	92.6
	500	474.5	94.8
Coffee	50	46.2	92.4
	200	188.6	94.3
	500	460.7	91.5
